# Supplementary material for: Genome-Wide Identification of DlGRAS Family and Functional Analysis of DlGRAS10/22 Reveal Their Potential Roles in Embryogenesis and Hormones Responses in Dimocarpus longan
Source: Int J Mol Sci. 2025 Oct 23;26(21):10323. doi: 10.3390/ijms262110323 (PMC12607546; doi:10.3390/ijms262110323)
Supplement: Supplementary file 1 [file ijms-26-10323-s001.zip › ijms-3875083-supplementary.pdf]

Appendix A

Genome-wide identification of DlGRAS family and functional analysis of *DlGRAS10/22* reveal their potential roles in embryogenesis and hormones responses in *Dimocarpus longan*

Guanghui Zhao[+], Mengjie Tang[+], Wanlong Wu, Wei Gao, Jinbing Xie, Jialing Wang, Zhongxiong Lai, Yuling Lin *[,‡] and Yukun Chen *[,‡]

（Institute of Horticultural Biotechnology, Fujian Agriculture and Forestry University, Fuzhou, Fujian, China, 350002）

Correspondence: Yuling Lin: buliang84@163.com; Yukun Chen: cyk68@163.com
+ These authors contributed equally to this work.
‡ These corresponding author contributed equally to this work.

Table S1 Basic information of DlGRAS family

Third generation gene ID	Gene Name	Number of amino acids	pI	Molecular weight	Instability index	Grand average of hydropathicity	Signal peptide	THM	Subcellular localization
Dlo001023	*DlGRAS1*	525	5.41	57490.43	43.58	-0.055	NO	0	Nuclear
Dlo001919	*DlGRAS2*	439	5.37	49381.77	51.97	-0.371	NO	0	Chloroplast
Dlo003348	*DlGRAS3*	599	4.92	67670.85	47.84	-0.254	NO	0	Nuclear
Dlo008128	*DlGRAS4*	453	6.18	50961.85	49.06	-0.142	NO	0	Nuclear
Dlo008385	*DlGRAS5*	429	5.64	48607.98	60.44	-0.332	NO	0	Chloroplast
Dlo008688	*DlGRAS6*	580	5.51	64305.53	51.69	-0.337	NO	0	Chloroplast
Dlo010756	*DlGRAS7*	747	5.64	83898.60	42.43	-0.472	NO	0	Nuclear
Dlo012001	*DlGRAS8*	602	6.09	68656.27	43.21	-0.259	NO	0	Cytoplasmic
Dlo012002	*DlGRAS9*	550	5.70	62939.07	43.53	-0.277	NO	0	Cytoplasmic
Dlo012500	*DlGRAS10*	411	5.74	46418.00	42.51	-0.168	NO	0	Cytoplasmic
Dlo012958	*DlGRAS11*	726	5.79	80134.38	56.76	-0.181	NO	0	Nuclear
Dlo012959	*DlGRAS12*	755	5.48	82489.97	56.62	-0.271	NO	0	Nuclear
Dlo014370	*DlGRAS13*	488	5.42	54158.92	36.72	-0.245	NO	0	Cytoplasmic

Dlo014371	*DlGRAS14*	493	5.31	54125.77	35.65	-0.218	NO	0	Cytoplasmic
Dlo014656	*DlGRAS15*	587	4.83	66230.75	53.76	-0.397	NO	0	Chloroplast
Dlo014938	*DlGRAS16*	458	6.53	52606.99	38.94	-0.401	NO	0	Nuclear
Dlo015668	*DlGRAS17*	604	4.98	66727.83	56.74	-0.288	NO	0	Nuclear
Dlo015697	*DlGRAS18*	572	5.84	63548.12	48.20	-0.129	NO	0	Nuclear
Dlo015987	*DlGRAS19*	454	5.62	50779.98	44.55	-0.112	NO	0	Chloroplast
Dlo016011	*DlGRAS20*	493	5.24	55647.35	51.49	-0.396	NO	0	Nuclear
Dlo016736	*DlGRAS21*	500	4.62	56956.92	48.70	-0.19	NO	0	Nuclear
Dlo017121	*DlGRAS22*	382	5.79	42050.45	39.45	0.057	NO	0	Cytoplasmic
Dlo017969	*DlGRAS23*	580	5.19	64100.41	46.25	-0.251	NO	0	Nuclear
Dlo019079	*DlGRAS24*	474	5.86	53309.53	50.39	-0.128	NO	0	Cytoplasmic
Dlo019330	*DlGRAS25*	505	4.98	56839.98	47.91	-0.363	NO	0	Cytoplasmic
Dlo019570	*DlGRAS26*	628	6.54	68520.14	55.77	-0.387	NO	0	Chloroplast
Dlo020862	*DlGRAS27*	574	5.02	62573.58	53.71	-0.267	NO	0	Nuclear
Dlo021157	*DlGRAS28*	539	5.50	60395.98	43.03	-0.368	NO	0	Nuclear
Dlo025134	*DlGRAS29*	473	6.15	53277.33	51.76	-0.183	NO	0	Nuclear
Dlo026427	*DlGRAS30*	761	5.21	86620.45	46.85	-0.527	NO	0	Nuclear
Dlo026428	*DlGRAS31*	644	4.75	71094.14	44.26	-0.504	NO	0	Nuclear
Dlo026429	*DlGRAS32*	646	6.70	74154.95	52.00	-0.325	NO	0	Chloroplast
Dlo026430	*DlGRAS33*	634	5.88	72023.41	46.33	-0.47	NO	0	Nuclear
Dlo026431	*DlGRAS34*	635	5.93	71771.96	50.11	-0.516	NO	0	Nuclear
Dlo026432	*DlGRAS35*	718	5.96	81077.24	46.31	-0.558	NO	0	Nuclear
Dlo026658	*DlGRAS36*	517	5.21	58719.24	47.64	-0.195	NO	0	Nuclear
Dlo026964	*DlGRAS37*	541	5.59	60971.83	52.57	-0.372	NO	0	Nuclear
Dlo027178	*DlGRAS38*	442	5.38	48869.75	44.03	-0.058	NO	0	Chloroplast
Dlo027543	*DlGRAS39*	668	5.65	73748.31	59.88	-0.271	NO	0	Nuclear
Dlo028988	*DlGRAS40*	520	4.86	58595.42	47.27	-0.282	NO	0	Nuclear
Dlo029236	*DlGRAS41*	750	5.85	84679.45	52.52	-0.495	NO	0	Chloroplast
Dlo029987	*DlGRAS42*	413	5.52	45609.03	53.43	-0.128	NO	0	Nuclear
Dlo030187	*DlGRAS43*	422	6.61	47674.76	45.15	-0.198	NO	0	Nuclear

Dlo030560	*DlGRAS44*	754	5.77	82713.96	55.40	-0.348	NO	0	Nuclear
Dlo031536	*DlGRAS45*	672	5.60	74405.43	56.01	-0.436	NO	0	Chloroplast
Dlo031697	*DlGRAS46*	721	5.9	80276.39	52.70	-0.34	NO	0	Nuclear
Dlo031902	*DlGRAS47*	518	6.26	57595.06	48.77	-0.094	NO	0	Cytoplasmic
/	/	212	5.63	24281.44	40.54	-0.595	NO	0	Cytoplasmic
/	/	545	5.35	61390.79	54.89	-0.245	NO	0	Nuclear

'/' indicates that there is no corresponding relationship in the second generation genome of longan

Table S2 Analysis of transcription start sites of *DlGRAS10* and *DlGRAS22* promoter

Gene name	Score	Position	Core promoter sequence	Transcription initiation site
DlGRAS10	1	790-840	ATATATATATATATATATATCTCATGAGAAGTATGCAGC CACAAGACACT	A
	0.94	866-916	TAGAAGTAAATATATAATAGGATGGATCATTATGCTATA TATCTTCGATT	A
	0.84	1403-1453	CGGATAAGAATATATGAGAGGGTATCATATTTTTCTACA AAACCAACACA	A
	0.91	1716-1766	TTAACTTCTTGTATATGTCATGGCTATGCCGGCCGTGGTC ATGGATTAAA	A
	0.95	1751-1801	TGGTCATGGATTAAAAAACTGCAAAAAAAACCTGTAATCT AAGCTAATGAG	A
	0.91	1853-1903	CATCTTTGCCTATAAACCTCCCCTTTAAAAACGTTCTCCA CCTCTTCTTG	C
	0.91	1868-1918	ACCTCCCCTTTAAAAACGTTCTCCACCTCTTCTTGACACC CTCTCACCAC	C
DlGRAS22	0.9	41-91	AGGATTGGGGTATAACGTTGGTGCATATGCTGCTGACGT GTGCCGGGTTG	T
	0.94	123-173	GCTTGTTCAAGATATAAAAGGCTTGTTGTTAACACGAGT CAATCCTGGTT	A

Table S3 The primers of *DlGRAS*

Primer Name	Primer sequence（5' to 3'）	Use
DlGRAS6-qF	CGTTGAAATCGAGGATGAAT	
DlGRAS6-qR	ATGAAACCCAGCTTGAAACA	
DlGRAS10-qF	CTCTCTTCCACATCCTCGCT	
DlGRAS10-qR	TCTTGGCTATGGGGTGGAAC	
DlGRAS12-qF	CGGCTCAATTTTGGACCC	
DlGRAS12-qR	CTCCCATTGCTTGCCTTTG	
DlGRAS15-qF	TTGCATGTGAGGGAGAGGAG	
DlGRAS15-qR	CCACCCAAAATGCAGCAG	
DlGRAS22-qF	CGTCAACTCGGTCATGCATT	
DlGRAS22-qR	GTGAACAACCTTCCTCCAGC	
DlGRAS23-qF	GCCATGACGGAGGTGTACTT	
DlGRAS23-qR	ACTGAGCGTTTCATGCCTCT	
DlGRAS26-qF	CGTTGAAATCGAGGATGAAT	qRT-PCR
DlGRAS26-qR	ATGAAACCCAGCTTGAAACA	
DlGRAS27-qF	TCAACAACAACAGCAACAAATG	
DlGRAS27-qR	GATCCGGTTTTGATTCGTTT	
DlGRAS28-qF	TGAGCAAGAATCCAAAACCA	
DlGRAS28-qR	ATTTCGCGAGAAGTTCATGT	
DlG1D1-qF	CACGTCTCAATTTCGGGTTTTAAA	
DlG1D1-qR	TCTTCCATAATTTATCGGTTCGCT	
DlGA20ox-qF	TGAACACTGTGAAGGAAAGGTTT	
DlGA20ox-qR	TCATTCACAAGCTCATCTAATGGT	
DlLEC1-qF	AACGCAAGACCATCACCTC	
DlLEC1-qR	AACCTTACCACAATCACCG	
DlLEC2-qF	ACCCACCAACCACAGTAGCA	
DlLEC2-qR	TGATGGAGTGTTTGAGCCG	

DlFUS3-qF	GGAGACTTTGTTAGCACCCA	
DlFUS3-qR	CTGTCCGAAGCCAATGTT	
DlABI3-qF	AGCCCAAACCCTAACCACA	
DlABI3-qR	TGCCAAAGAGTCACCCGTA	
NbGUS-qF	CGACTGGGCAGATGAACAT	
NbGUS-qR	ATACTCCACATCACCACGCT	
DlUBQ-qF	GCCGACTACAACATCCAGAAG	
DlUBQ-qR	GCTTGGTGTAGGTCTTCTTCTT	
DlEF1α-qF	GATAATTCCCACCAAGCCCAT	
DlEF1α-qR	GGGTCCTTCTTCTCAACACTCT	qRT-PCR (Reference gene)
DlACTB-qF	TGCTATCCTTCGGTTGGACC	
DlACTB-qR	CGGACGATTTCCCGTTCAG	
Nb18S-qF	CCTGAGAAACGGCTACCACAT	
Nb18S-qR	CACCAGACTTGCCCTCCA	
DlGRAS10-F	GAAGATCTATGATGAAAGGAGGTTTTGAAG	
DlGRAS10-R	GACTAGTAATGTAGGTAGAGTAGTAATGTAAGGC	Construction of DlGRAS:GFP
DlGRAS22-F	CATGCCATGGATGGGAGAGGGGGTTAGC	
DlGRAS22-R	GAAGATCTGTAGTGTGAGTGATGATTGACTTG	
DlGRAS10-F	AACTGCAGATGATTGAGTTGGGTGAGATGAGT	
DlGRAS10-R	CATGCCATGGCGAGGTTTTCTTCATCACTGCTAGT	Construction of DlGRAS-pro:GUS
DlGRAS22-F	CGGGATCCTTGATGCATATATGACAAGAAGGAAC	
DlGRAS22-R	CATGCCATGGCCGTGATGCAGATAGAAAAAGAG	
DlGRAS10-F	GGGGTACCATGATGAAAGGAGGTTTTGAAGT	
DlGRAS10-R	CGGGATCCTCAAATGTAGGTAGAGTAGTAATGTAAGGC	Construction of DlGRAS:GUS
DlGRAS22-F	GGGGTACCATGGGAGAGGGGGTTAGC	
DlGRAS22-R	AACTGCAGTTAGTAGTGTGAGTGATGATTGACTTG	
